# Improve Performance of Soy Flour-Based Adhesive with a Lignin-Based Resin

**DOI:** 10.3390/polym9070261

**Published:** 2017-07-03

**Authors:** Xiaochun Zhang, Yuding Zhu, Youming Yu, Jiangang Song

**Affiliations:** 1 School of Engineering, Zhejiang Agriculture and Forestry University, Lin’an 311300, China; zhuyuding-107@163.com (Y.Z.); yuyouming@zafu.edu.cn (Y.Y.); 2 Zhejiang Yongyu Bamboo Joint-Stock Co., Ltd., Anji 313301, China; sjgcw@163.com

**Keywords:** soy flour, lignin, adhesive, plywood, bond strength

## Abstract

A lignin-based resin (LB) was used to improve the performance of soy flour-based adhesives. Soy flour (SF), polyamidoamine-epichlorohydrin (PAE), and LB were used to develop a plywood adhesive. The solid content and viscosity of the adhesive, the functional groups, the thermo-stability, and the crystallinity of the cured adhesives were characterized, and the performance of the resultant adhesive was evaluated by fabricating three-ply plywood. Results showed that the LB and PAE mixture used to modify the SF adhesive improved both dry and wet bond strength by 66.3% and 184.2%, respectively. Therefore, the PAE improved the wet bond strength, and the LB improved the dry bond strength. The improvement was attributed to: (1) the reaction of LB/PAE with the functions of the soy protein to form a cross-linking network; (2) a polycondensation reaction between the LB molecules improved the crosslinking density of the adhesive to form an interpenetration structure with cross-linked proteins; and (3) the easy penetration of the LB into the wood surface that enhanced interlocking between the wood and adhesive. Furthermore, the denser structure created by the LB and the PAE mixture improved thermal stability and decreased the crystallinity of the cured adhesive. The use of the LB and the PAE mixture increased the solid content by 35.5%, while still making its viscosity acceptable for industrial applications.

## 1. Introduction

Formaldehyde-based resins—particularly melamine-urea-formaldehyde resin—are widely used to fabricate plywood panels [1]; however, they are non-biodegradable and petroleum-derived, resulting in environmental concerns regarding their preparation and use [2,3]. Thus, the development of adhesives based on ecological and renewable natural resources is crucial. Soy flour has been used as a wood adhesive for decades, as it is a renewable, abundant, readily available, and inexpensive raw material [4]. Nevertheless, its poor water resistance has limited the application of soy flour-based adhesives. Many attempts have been made to improve the water resistance of soy flour-based adhesives, including protein denaturing agent modification and crosslinker modification. Denaturing agents such as alkalis [5], urea [6], and sodium dodecyl sulfate (SDS) [7] can unfold protein molecules to expose their internal hydrophobic groups that improve the water resistance of the adhesive. However, the water resistance of the resultant plywood does not meet the requirements for interior use. Crosslinker modifications such as polyamidoamine-epichlorohydrin (PAE) resin [8], glycidyl methacrylate [9], and polyethylene glycol diacrylate [10] can cross-link the protein molecules by reacting with the functions to form a network that improves the water resistance of the adhesive. PAE has been shown to be the most effective crosslinker in improving the water resistance of the adhesive; however, soy flour-based adhesives with those crosslinkers have a low dry bonded strength, which limits their application—particularly in the secondary operation process of its resultant plywood. This is due to large protein molecules that make it difficult to penetrate the wood surface to form an interlock. Thus, it is important to improve the performance of soy flour-based adhesives in both dry and wet states.

The use of synthetic resins such as melamine-urea-formaldehyde (MUF) [11], phenol formaldehyde (PF) [12], or latex-based resins [13,14] can improve the water resistance of the adhesive. Nevertheless, these adhesives have issues; for example, MUF resins result in formaldehyde emissions, and latex resins have a low water resistance. PF resin effectively improves both the bond strength and water resistance of the adhesive, but it has disadvantages such as a high pH value, which causes a degradation of the soy protein and leads to a decrease in bond strength. The weight ratio of protein/PF in the adhesive is more than 1:1, therefore making the soy flour more like a filler in the PF resin, and not a soy protein-based adhesive. The curing temperature of PF resin is also high (≥150 °C), and the raw material of PF resins is derived from fossil fuels and non-renewable resources.

In this study, a soy flour (SF)-based adhesive was synthesized using soy flour and PAE. Lignin was treated with phenol and formaldehyde to develop a lignin resin (LB) with phenolic hydroxyl methylation, which acted as a modifier to improve the bond performance of the adhesive. Different additions of LB resin were used to develop different adhesives. The performance of the resultant adhesives, including the solid content and viscosity of the adhesive, as well as the functional groups, thermostability, and fracture surface of the cured adhesive were characterized. Three-ply plywood was fabricated with the resultant adhesives, and their wet bond strength was tested.

## 2. Experimental Methods

### 2.1. Materials

Soy flour (200 mesh) is the meal after removing the oil from soybeans, and was obtained and milled to flour (SF) from Fuda Protein Biotech Company in Hang Zhou, China. Components of the soy flour were: 52.2% soy protein, 38.2% saccharides, 7.5% moisture, 2.6% ash, and 0.5% fat. AR-grade reagents of phenol, formaldehyde solution (37–0 wt %) and sodium hydroxide were obtained from Zhejiang Chemical Reagent Co., Hang Zhou, China. The lignin (from soft wood) was produced by the Yongtai Paper Co., Ltd. (Hang Zhou, China), and contained 82.5% klason lignin, 6.2% acid-soluble lignin, 2.5% ash, 5.1% sugar, and 3.7% others. PAE was obtained from Kaiyuan Chemical Ltd. (Hang Zhou, China), where the solid content was 12.5%, with a viscosity ranging from 25–45 mPa·s. Eucalyptus veneer (60 × 60 × 1.7 cm, 5% of moisture content) was provided by Jiashan, China.

### 2.2. Lignin-Based Resin (LB) Preparation

The LB resin was synthesized by batch copolymerization using lignin, phenol, and formaldehyde. In the first step, lignin (260 g) and phenol (260 g) with formaldehyde (400 g, 37 wt %) and NaOH (100 g, 50 wt %) were mixed and stirred in a flask. The mixture was heated to 80 °C and maintained for 1 h. Next, the second portion of formaldehyde (300 g, 37 wt %) and NaOH (80 g, 50 wt %) was added to the flask and stirred for 1 h at 80 °C. The mixture was cooled to 40 °C and the free formaldehyde was removed through a vacuum distillation process to obtain the LB resin. The solid content of the resultant resin was 62.2%, and the viscosity was 1080 mPa·s at 20 °C.

### 2.3. Adhesive Preparation and Measurement

Soy flour was mixed with water and stirred for 15 min at 30 °C for each adhesive sample. LB and PAE were then added and stirred for an addition 15 min at 30 °C. The different adhesive formulations are shown in Table 1.

#### 2.3.1. Solid Content Measurement

The adhesive solid content was measured by placing approximately 3 g (α) of the adhesive in the oven and drying at 102 °C for 2 h to obtain a weight (β). The value of the solid content was calculated using the following equation. The average value of the solid content was calculated over six parallel samples.

(1) $$\mathrm{Solid}\;\mathrm{Content}\;(\%)=\frac{\beta \;(g)}{\alpha \;(g)}\times 100\%$$

#### 2.3.2. Dynamic Viscoelastic Measurement

The apparent viscosity of different adhesives was determined by a Brookfield viscometer with a spinning rate of 1 rpm, where the averages were determined over 10 measurements taken in 3 min increments at 30 °C.

#### 2.3.3. Fourier Transform Infrared (FTIR) Spectroscopy

The adhesive was cured in an oven at 130 °C for 1 h then ground into a 150-mesh powder by grinding pellets in an agate mortar. The Fourier transform infrared (FTIR)spectra of the cured adhesive were tested using a Nicolet 7600 spectrometer (Nicolet Instrument Corporation, Madison, WI, USA) with an attenuated total reflectance (ATR) accessory (a single reflection diamond crystal) from 500–4000 cm^−1^ with a 4 cm^−1^ resolution over 16 scans.

#### 2.3.4. Thermogravimetric (TG) Measurement

The adhesive was cured in an oven at 130 °C for 1 h then ground into a 150-mesh powder. The thermal stability of the cured adhesive samples was tested using a thermal gravimetric analyzer (TGA) instrument (TA Q50, WATERS Company, New Castle, DE, USA). The 3 mg powdered samples were weighed in a platinum cup and scanned from 30–600 °C at a heating rate of 10 °C/min in a nitrogen environment. The weight change was recorded throughout.

#### 2.3.5. X-ray Diffraction (XRD) Measurement

The adhesive was cured in an oven at 120 ± 2 °C until the weight remained constant. It was then ground into a powder. X-ray diffraction (XRD) patterns were recorded on a D8 advance diffractometer (Bruker, Madison, WI, USA) using a cobalt source and 0.02 theta scan ranging from 5° to 60° at 45 kV and 30 mA. The index of the sample determination was carried out using a Jade 5.0 program, Bruker, Madison, WI, USA.

### 2.4. Plywood Preparation and Testing

The three-ply plywood was fabricated using the following conditions: 130 °C hot pressing temperature, 90 s/mm hot pressing time, 200 g/m^2^ glue spreading, and 0.8 MPa hot pressing pressure.

The wet bond strength was measured via wet bond strength test in accordance with the description in the China National Standards (GB/T 17657-2013). Twenty plywood specimens (2.5 cm × 10 cm) were cut from two parallel plywood panels and submerged into water at 63 ± 2 °C for 3 h before a tension test. The wet bond strength was calculated by the following equation:

(2) $$\mathrm{Bondingstrength}(\mathrm{MPa})=\frac{\mathrm{TensionForce}(N)}{{\mathrm{Gluingarea}(m}^{2})},$$

## 3. Results and Discussion

### 3.1. Solid Content Measurement

The solid content of the adhesive was calculated from three parallel samples as seen in Table 2. When the wood adhesive was used, the adhesive with a high solid content had superior performance [15]. As soy protein-based adhesives are water-based with a low solid content (meaning that more water is used in the adhesive formulation), this led not only to a low adhesive spread, but the water also needed to be removed during the hot press process, which costs energy and increases the interior force of the resultant plywood. Adhesive a exhibited a solid content of 28.2%, where at this level of solid content, the adhesive had flow issues, indicating that the additives determined the increase in the solid content. When mixed with LB resin, the solid content of Adhesive b reached 32.9%, which was an increase of 16.7% when compared to Adhesive a. After the addition of PAE, the solid content of Adhesive d increased to 35.5%, which was an increase of 25.9% when compared to Adhesive a. However, when only PAE and the SF adhesive were mixed together, the solid content of Adhesive c was 31.2%, which was a decrease of 12.1% when compared to Adhesive d. According to the literature, the solid content of soy protein-based adhesives has ranged from 32–36% [16,17]. Based on those results, the solid content of Adhesive d met the requirements for the plywood adhesive application.

### 3.2. Dynamic Viscoelastic Measurement

The viscosity of the adhesives is shown in Table 3. The viscosity of the adhesive increased from 28,510 to 687,500 mPa·s after the addition of LB (Adhesive b). This was thought to have occurred as the LB contained free sodium hydroxide (NaOH), which is effective at denaturing proteins, so it unfolded the soy protein molecules and exposed the inner functional groups [18]. This increased the friction between the protein molecules, resulting in an increase in adhesive viscosity. After the addition of PAE, the viscosity of the resultant adhesive decreased from 28,510 to 19,840 mPa·s, which may be due to the low viscosity of PAE itself. In addition, the PAE contains smaller molecules than the soy protein which act as a molecular lubricant that reduces friction between the molecules, resulting in decreases to adhesive viscosity. This was consistent with results obtained by Yuan [19]. The viscosity of Adhesive d was 40% lower than that of Adhesive b, further substantiating that PAE had a viscosity reduction effect.

### 3.3. FTIR Spectroscopic Analysis

**Figure 1:**
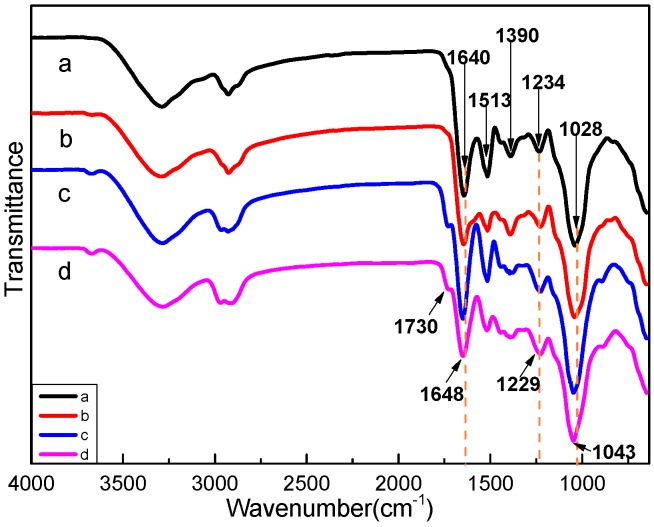
Fourier transform infrared (FTIR) spectra of the different adhesives: (**a**) SF adhesive; (**b**) SF/LB adhesive; (**c**) SF/PAE adhesive; and (**d**) SF/LB/PAE adhesive.

**Scheme 1:**
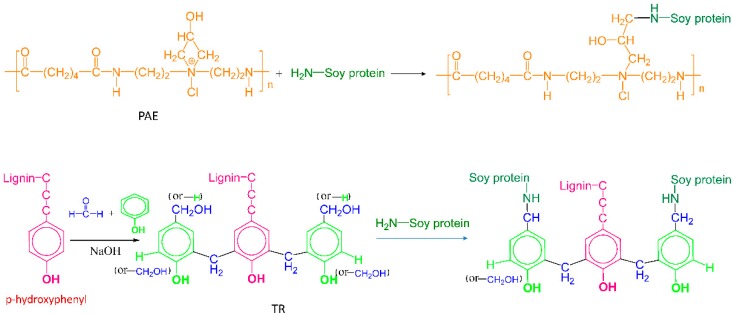
The reaction of the adhesive components. (PAE: polyamidoamine-epichlorohydrin, TR: Tannin based resin).

The FTIR spectra of the various adhesives are shown in Figure 1. Peaks of Adhesive a—observed at 1640, 1513, and 1234 cm^−1^—were attributed to the characteristic peaks of peptide, amide I (C=O stretching), amide II (N–H bending), and amide III (C–N and N–H stretching), respectively [20]. The peaks at 1390 and 1028 cm^−1^ were attributed to COO– and C–O bending, respectively [21], which was considered constant across all adhesive formulations. After the addition of LB, the amide II (1513 cm^−1^) of Adhesive b decreased when compared with Adhesive a (referring to the peak at 1390 cm^−1^), indicating that N–H decreased in Adhesive b, which was due to LB reacting with the N–H groups on the soy protein. This reaction formed a cross-linked network in the adhesive to enhance the water resistance of the adhesive. In the spectra of Adhesive b, the absorption peak of C=O stretching (amide I) and C–O bending moved from 1640 to 1648 cm^−1^ and 1028 to 1043 cm^−1^ (blue shift), respectively, when compared to Adhesive a, suggesting a denser structure was formed in the adhesive, which further improved the water resistance. When soy protein molecules are cross-linked to form a network, the structure becomes dense and ordered, resulting in the vibration of functional groups needing more energy, which presents a blue shift [22].The same change was observed in the spectra of Adhesive c, indicating that PAE also reacted with the –NH groups on the soy protein molecule and formed a denser crosslinking structure. When mixing LB and PAE as the modifier, the absorption peak of –NH_2_ further decreased, suggesting a much denser structure formed in the adhesive, thus further increasing the water resistance of the adhesive. The specific reaction is discussed in Scheme 1.

### 3.4. TGA Analysis

**Figure 2:**
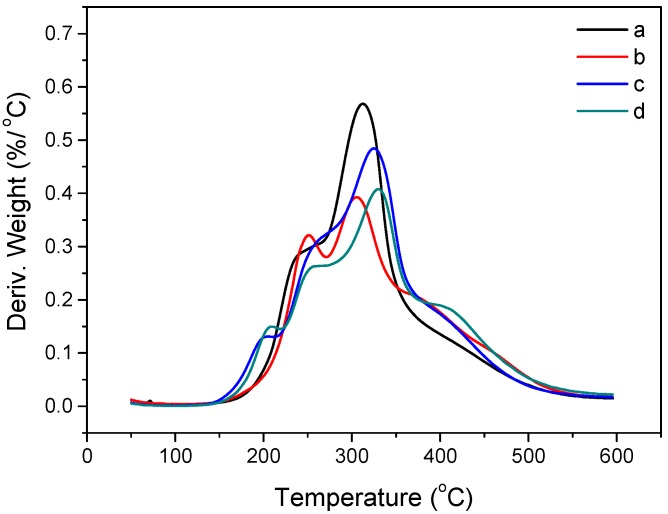
The derivative thermogravimetric (DTG) curves of the different adhesives: (**a**) SF adhesive; (**b**) SF/LB adhesive; (**c**) SF/PAE adhesive; and (**d**) SF/LB/PAE adhesive.

**Scheme 2:**
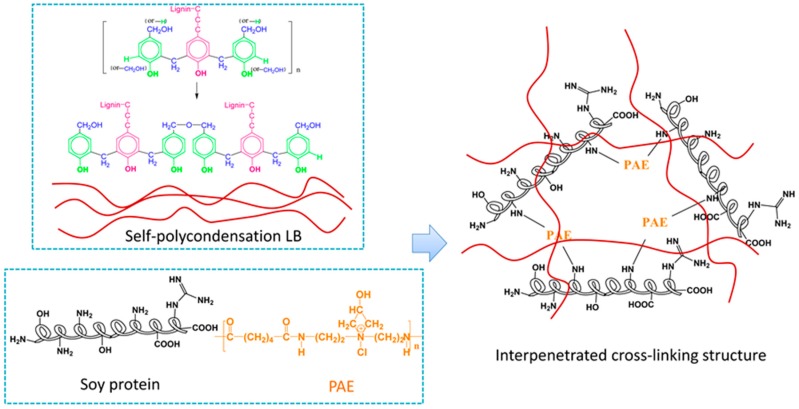
The formation of the interpenetrated network.

The derivative thermogravimetric (DTG) curves of the different adhesives are shown in Figure 2. According to Liu [22], the thermal degradation process of a soy protein-based adhesive can be divided into three main stages: the first stage(weight loss of 0–100 °C) was attributed to the evaporation of residual moisture; the second stage (weight loss of 100–270 °C) was due to unstable chemical bond decomposition; and in the third stage (from 270–500 °C), the main structure of the adhesive degrades and the covalent bonding in the structure decomposes. For Adhesive a, two peaks were observed in the second stage, indicating the existence of unstable bonds in the cured adhesive. After mixing PAE with Adhesive a, the peak at the second and third stage decreased, indicating that PAE reacted with the protein molecule to form stable chemical bonds. This was a different structure compared with Adhesive a, and increased thermo-stability in the resulting adhesive. Theoretically speaking, LB had a similar reaction with protein based on the FTIR analysis, which presented a similar thermal behavior. However, the addition of LB in the adhesive formulation (Adhesives a and d) presented obvious peaks at 220–260 °C, indicating an increase in unstable bonds in Adhesive c when compared to the adhesive without LB. This was attributed to the unstable ether bond formation by a self-condensation reaction of LB. This linked LB penetrated with the cross-linked soy protein to form an interpenetration network, further increasing the water resistance of the resulting adhesive (Scheme 2).

### 3.5. XRD Analysis

The crystallinity of the different adhesive formulations is presented in Table 4. The crystallinity of Adhesive a was 15.5%, as a cross-linked structure formation in an adhesive reduces its crystallinity within the wood adhesive. Crystallinity decreased to 14.6% after adding LB, most likely due to the reaction between the LB and soy protein. This also increased the cross-linking density of the cured adhesive. Furthermore, crystallinity decreased to 13.1% after adding PAE, which was 10.3% lower than Adhesive b, indicating that a denser structure was formed in Adhesive c that led to an increase in water resistance. This was in agreement with the results of the wet bond strength measurements. Crystallinity further decreased to 11.6% after the addition of LB and PAE, indicating a more compact structure formation in the cured Adhesive d. This was attributed to the reaction between the protein and LB/PAE that created a cross-linking structure. In addition, the compact structure also reached the interpenetration network formation by LB condensation, which further increased the density degree of the adhesive. The results of the adhesive crystallinity analysis were in accordance with the results found in the FTIR and TGA analyses.

### 3.6. Dry Bond Strength

**Figure 3:**
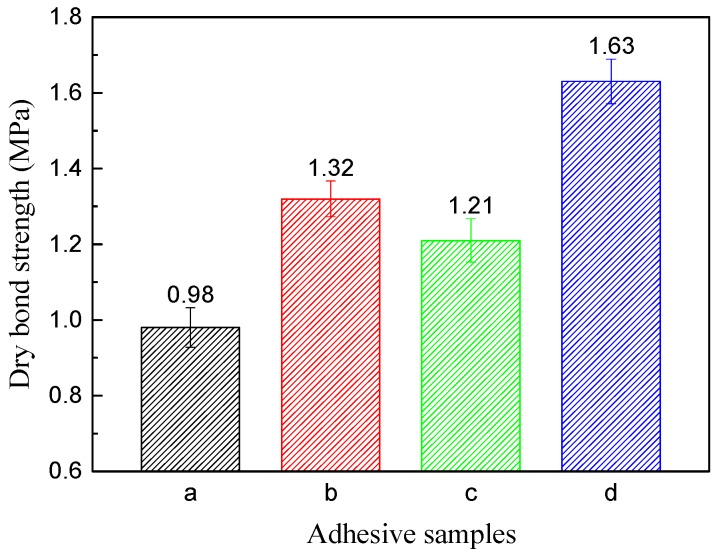
The dry bond strength of the plywood with the different adhesives: (**a**) SF adhesive; (**b**) SF/LB adhesive; (**c**) SF/PAE adhesive; and (**d**) SF/LB/PAE adhesive.

The dry bond strength of the plywood bonded with different adhesives is shown in Figure 3. The dry bond strength of the plywood bonded with Adhesive a was 0.98 MPa, as the large protein molecules made it difficult for the adhesive to penetrate into the wood surface to form a mechanically interlocked structure, resulting in a low dry bond strength [14]. The interaction between the soy protein molecules and wood was low, further reducing the bond performance of the adhesive. The dry bond strength of the resultant plywood increased to 1.32 MPa after adding LB, as the LB molecules are smaller and can more easily form mechanical interlocking, thus improving the dry bond strength. In addition, the LB reacted with the protein molecules and themselves to form a crosslinking structure that increased cohesive strength and improved bond strength. Analogous results were found with Adhesive c. With the addition of PAE to the adhesive formulation, the PAE cross-linked the protein molecules to form denser structures that improved bond strength. However, PAE contributed less in forming mechanically interlocked structures, so improvement of the plywood with Adhesive c was lower than that of Adhesive b. When mixing PAE and LB, the dry bond strength of plywood increased to 1.63 MPa, which was 66.3% higher than Adhesive a. This improvement was attributed to the small molecules increasing interlocking between the wood and adhesive. The strength of the adhesive itself was enhanced as chemical reactions occurred between the phenolic hydroxyl methyl (adhesive) and hydroxyl (wood), thus increasing the interaction forces between the wood and adhesive [23].

### 3.7. Water Shear Strength

**Figure 4:**
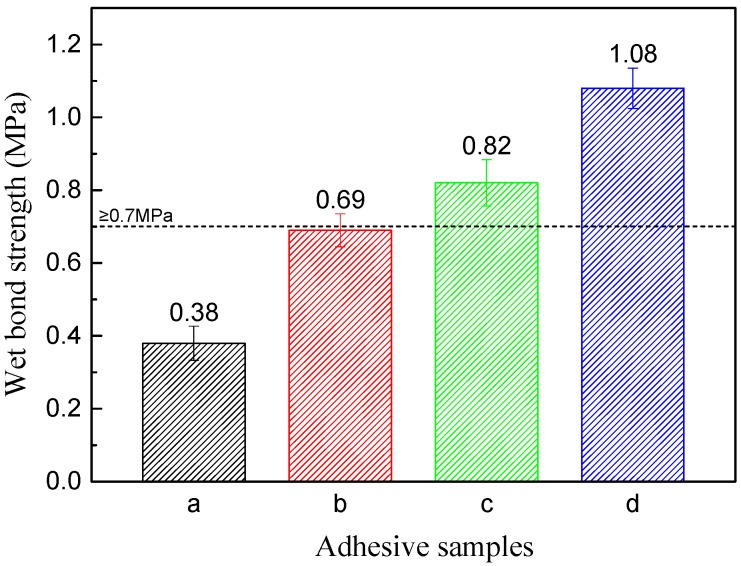
The wet bond strength of the plywood with different adhesives: (**a**) SF adhesive; (**b**) SF/LB adhesive; (**c**) SF/PAE adhesive; and (**d**) SF/LB/PAE adhesive.

The wet bond strengths of the plywood bonded by the various adhesives are shown in Figure 4. The wet bond strength of the plywood bonded with Adhesive a was 0.38 MPa, as its curing process involved the loss of water and molecules twined with each other. During this curing process, the main forces inside Adhesive a were hydrogen bonds, which were easily broken from moisture intrusion [24]. When using LB, the wet bond strength of plywood with Adhesive b reached 0.69 MPa. This improvement was caused by many reasons. As NaOH in LB is an effective denaturing agent that unfolds the protein molecules to expose the hydrophobic groups, this resulted in an improvement in adhesive water resistance [25]. The phenolic hydroxyl group on LB reacted with the functions of protein (such as, –NH_2_, –COOH), and formed a cross-linked network, further increasing the water resistance of the adhesive. The PAE-modified SF adhesive also increased the wet bond strength of plywood with Adhesive c by 115.8% to 0.82 MPa. In effect, PAE reacted with the functions of the protein to form a denser structure to enhance the water resistance of the adhesive, thus demonstrating its efficacy as a curing agent of soy protein. After mixing PAE and LB, the wet bond strength of the plywood was 1.08 MPa, an increase of 184.2% when compared with Adhesive a. The NaOH in LB unfolded the soy protein molecule and exposed the active groups, which facilitated the reaction between the protein molecules and PAE. In addition, the LB reacted with itself through a polycondensation reaction to form a network (TGA analysis) that penetrated the PAE cross-linked soy protein network to form an interpenetrating network, therefore improving the water resistance of the resultant plywood.

## 4. Conclusions

Using LB and PAE to modify SF adhesives improved both the dry and wet bond strength by 66.3% and 184.2%, respectively. The PAE enhanced the wet bond strength, and the LB enhanced the dry bond strength. The improvement of the wet bond strength was attributed to: (1) the reaction of LB and PAE with the functions of the soy proteins to form a cross-linking network; and (2) the polycondensation reactions between the LB molecules further improved the crosslinking density of the adhesive and formed an interpenetration structure with cross-linked proteins. The improvement in dry bond strength was attributed to LB easily penetrating the wood surface to form more interlocks with the wood. Furthermore, the cross-linked and interpenetration structure created by LB and PAE also improved thermal stability and decreased the crystallinity of cured adhesives. The addition of the LB and the PAE mixture increased the solid content by 35.5%, resulting in the adhesive obtaining an acceptable viscosity for industrial applications. Thus, this appears to be an effective way to promote soy protein adhesive applications.

## Figures and Tables

**Table 1:** Adhesive formulations. PAE: polyamidoamine-epichlorohydrin; LB: lignin-based resin; SF: soy flour.

Adhesive	Sample	Formulation
a	SF adhesive	Soy flour (30 g); Water (70 g)
b	SF/LB adhesive	Soy flour (30 g); Water (70 g); LB (10 g)
c	SF/PAE adhesive	Soy flour (30 g); Water (40 g); PAE (30 g)
d	SF/PAE/LB adhesive	Soy flour (30 g); Water (40 g); PAE (30 g); LB (10 g)

**Table 2:** The solid content of the adhesives: (**a**) SF adhesive; (**b**) SF/LB adhesive; (**c**) SF/PAE adhesive; and (**d**) SF/LB/PAE adhesive.

Adhesive	a	b	c	d
solid content (%)	28.2	32.9	31.2	35.5

**Table 3:** The viscosity of the adhesives: (**a**) SF adhesive; (**b**) SF/LB adhesive; (**c**) SF/PAE adhesive; and (**d**) SF/LB/PAE adhesive.

Adhesive	a	b	c	d
Initial viscosity (mPa·s)	28,510	687,500	19,840	541,200

**Table 4:** The crystallinity of different cured adhesives: (**a**) SF adhesive; (**b**) SF/LB adhesive; (**c**) SF/PAE adhesive; and (**d**) SF/LB/PAE adhesive.

Adhesive	a	b	c	d
The crystallinity (%)	15.5	14.6	13.1	11.6
